# Supplementary material for: Seizure onset and offset pattern determine the entrainment of the cortex and substantia nigra in the nonhuman primate model of focal temporal lobe seizures
Source: PLoS One. 2024 Aug 28;19(8):e0307906. doi: 10.1371/journal.pone.0307906 (PMC11356443; doi:10.1371/journal.pone.0307906)
Supplement: S1 File — (DOCX) [file pone.0307906.s001.docx]

S1. Description of the staining method:

After perfusion, the brains were cut coronally into 30 mm thick blocks, and post-fixed overnight in 4% paraformaldehyde. The blocks were then cryoprotected in ascending sucrose solution (10, 20 and 30% in PBS), cut into 50 μm-thick coronal sections using a freezing microtome and collected in series. Sections encompassing the SN from one series were immunostained using a tyrosine hydroxylase antibody, a marker of dopaminergic neurons to identify the position of the SN. Sections were rinsed in phosphate buffered saline (PBS; P3813, Sigma Aldrich, St. Louis, MO), permeabilized in PBS containing 0.3% hydrogen peroxide (H1009, Sigma Aldrich) and 0.1% of Triton-X (T8787, Sigma Aldrich) for 20 minutes with no shaking. Following several PBS rinses, sections were then incubated in 4% normal goat serum/PBS (NGS; 017-000121; Jackson Immunoresearch Laboratories) for 30 minutes at RT with shaking, rinsed in PBS, and incubated overnight in rabbit anti-TH antibodies (cat#P40101-150, Pel-Freez, Rogers, AR) at 4C with shaking. The next day, sections were rinsed and incubated in biotin SP conjugated donkey anti-rabbit IgG antibodies (cat#711-065-152, Jackson Immunoresearch Laboratories) in PBS containing 2% NGS at RT for 1hr with shaking, followed by additional rinses and an overnight incubation in avidin biotin complex (Vectastain Elite ABC kit HRP, standard, cat#PK-6100, Vector Laboratories). The next day sections were rinsed in PBS and incubated in a solution containing 3’3’-diaminobezidine tetrahydrochloride hydrate (DAB; cat#D3737, Tokyo Chemical Industry) and hydrogen peroxide for 12 minutes at RT with shaking. Stained sections were mounted on gelatin coated glass slides, dried and coverslipped with Permount (SP15-100, Fischer Chemical). Sections through the SN from an adjacent series were mounted on glass slides and stained with cresyl violet to verify the electrode tract.
