# Supplementary material for: Seizure onset and offset pattern determine the entrainment of the cortex and substantia nigra in the nonhuman primate model of focal temporal lobe seizures
Source: PLoS One. 2024 Aug 28;19(8):e0307906. doi: 10.1371/journal.pone.0307906 (PMC11356443; doi:10.1371/journal.pone.0307906)
Supplement: S1 Table — (DOCX) [file pone.0307906.s002.docx]

S1 Table: Mean ± SEM values obtained in the pre-ictal, onset, offset and post-ictal periods in the HPC and SN for NHP1 and NHP 2. Statistical comparison performed with a Friedman repeated test and Dunnett’s for post hoc comparison with the values preceding the seizures, *<0.05, **<0.01, ***<0.001. Numbers in bold represent results consistent for both animals.

|  |  | NHP 1 | | | |  | NHP 2 | | | |
| --- | --- | --- | --- | --- | --- | --- | --- | --- | --- | --- |
|  |  | Pre-ictal | Onset | Offset | Post-ictal |  | Pre-ictal | Onset | Offset | Post-ictal |
| HPC | [1–7Hz] | 0.049±0.014 | **0.127±0.024 **** | 0.078±0.017 | 0.042±0.010 |  | 0.082±0.006 | **0.116±0.005***** | 0.069±0.004 | **0.049±0.002***** |
|  | [8–12Hz] | 0.010±0.001 | **0.060±0.008***** | **0.070±0.015***** | **0.038±0.008*** |  | 0.008±0.001 | **0.020±0.001***** | **0.018±0.001***** | 0.010±0.001 |
|  | [13–25Hz] | 0.003±0.001 | **0.030±0.003***** | **0.025±0.006**** | **0.054±0.012***** |  | 0.002±0.001 | **0.008±0.001***** | **0.005±0.001***** | 0.028±0.001 |
|  |  |  |  |  |  |  |  |  |  |  |
| SN | [1–7Hz] | 0.027±0.006 | 0.023±0.005 | 0.027±0.007 | 0.021±0.006 |  | 0.009±0.001 | 0.010±0.001 | 0.012±0.001 | 0.008±0.001 |
|  | [8–12Hz] | 0.004±0.001 | **0.006±0.001*** | 0.005±0.001 | 0.004±0.001 |  | 0.002±0.001 | **0.004±0.001***** | 0.003±0.001*** | 0.002±0.001 |
|  | [13–25Hz] | 0.001±0.001 | **0.003±0.001*** | 0.001±0.001 | 0.001±0.001 |  | 0.001±0.001 | **0.002±0.001***** | 0.001±0.001*** | 0.001±0.001 |
