# Supplementary material for: Seizure onset and offset pattern determine the entrainment of the cortex and substantia nigra in the nonhuman primate model of focal temporal lobe seizures
Source: PLoS One. 2024 Aug 28;19(8):e0307906. doi: 10.1371/journal.pone.0307906 (PMC11356443; doi:10.1371/journal.pone.0307906)
Supplement: S2 Table — (DOCX) [file pone.0307906.s003.docx]

S2 Table­: Mean ± SEM values of the HPC/SN coherence obtained before, at the beginning and the end of the seizure for NHP1 and NHP 2. Statistical comparison performed with a Friedman repeated test and Dunnett’s for post hoc comparison with the pre-ictal values, *<0.05, **<0.01, ***<0.001. Numbers in bold represent results consistent for both animals.

|  |  |  | NHP 1 | | | |  | NHP 2 | | | |
| --- | --- | --- | --- | --- | --- | --- | --- | --- | --- | --- | --- |
| HPC-SN |  | Pre-ictal | | Onset | Offset | Post-ictal |  | Pre-ictal | Onset | Offset | Post-ictal |
|  | [1–7Hz] | 0.55±0.05 | | 0.56±0.04 | 0.52±0.03 | 0.53±0.03 |  | 0.60±0.01 | 0.55±0.01* | 0.51±0.01*** | 0.53±0.01* |
|  | [8–12Hz] | 0.57±0.04 | | 0.57±0.03 | 0.55±0.03 | 0.60±0.03 |  | 0.53±0.01 | 0.48±0.01 | 0.47±0.01 | 0.50±0.01 |
|  | [13–25Hz] | 0.50±0.03 | | **0.55±0.02*** | 0.54±0.01* | 0.60±0.03* |  | 0.53±0.01 | **0.62±0.01***** | 0.53±0.01** | 0.50±0.01** |
