# Supplementary material for: Seizure onset and offset pattern determine the entrainment of the cortex and substantia nigra in the nonhuman primate model of focal temporal lobe seizures
Source: PLoS One. 2024 Aug 28;19(8):e0307906. doi: 10.1371/journal.pone.0307906 (PMC11356443; doi:10.1371/journal.pone.0307906)
Supplement: S3 Table — (DOCX) [file pone.0307906.s004.docx]

S3 Table: Mean ± SEM spectra values and coherence obtained in the pre-ictal and onset periods in the HPC and SN for LAF and HAS onset patterns. Statistical comparison performed with a Wilcoxon Signed Rank test for paired quantitative data, *<0.05, **<0.01, ***<0.001. Comparisons between LAF and HAS seizures were performed with a Mann-Whitney Rank Sum test (# <0.05). Statistical values were corrected for multiple comparison using Bonferroni correction.

|  |  | LAF (n=44) | |  |  | HAS (n=18) | |
| --- | --- | --- | --- | --- | --- | --- | --- |
|  |  | Pre-ictal | Onset |  |  | Pre-ictal | Onset |
| HPC | [1–7Hz] | 0.063±0.006 | 0.107±0.006 *** |  |  | 0.104±0.014# | 0.137±0.009**# |
|  | [8–12Hz] | 0.007±0.001 | 0.029±0.004*** |  |  | 0.010±0.001 | 0.023±0.002*** |
|  | [13–25] | 0.002±0.001 | 0.013±0.002*** |  |  | 0.003±0.001 | 0.009±0.001*** |
|  |  |  |  |  |  |  |  |
| SN | [1–7Hz] | 0.010±0.001 | 0.011±0.001 |  |  | 0.015±0.003 | 0.016±0.003 |
|  | [8–12Hz] | 0.002±0.001 | 0.004±0.001*** |  |  | 0.003±0.001 | 0.005±0.001* |
|  | [13–25Hz] | 0.001±0.001 | 0.002±0.001*** |  |  | 0.001±0.001 | 0.004±0.001***# |
|  |  |  |  |  |  |  |  |
| HPC/SN | [1–7Hz] | 0.56±0.02 | 0.54±0.01 |  |  | 0.68±0.03# | 0.59±0.02** |
|  | [8–12Hz] | 0.52±0.01 | 0.50±0.01 |  |  | 0.58±0.03 | 0.50±0.03 |
|  | [13–25Hz] | 0.55±0.01 | 0.59±0.01* |  |  | 0.63±0.02# | 0.63±0.02 |
