# Supplementary material for: Seizure onset and offset pattern determine the entrainment of the cortex and substantia nigra in the nonhuman primate model of focal temporal lobe seizures
Source: PLoS One. 2024 Aug 28;19(8):e0307906. doi: 10.1371/journal.pone.0307906 (PMC11356443; doi:10.1371/journal.pone.0307906)
Supplement: S4 Table — (DOCX) [file pone.0307906.s005.docx]

S4 Table: Mean ± SEM spectra values and coherence obtained in the offset and post-ictal periods in the HPC and SN for ARR, RHY and BS offset patterns. Statistical comparison performed with a Wilcoxon Signed Rank test for paired quantitative data, *<0.05, **<0.01, ***<0.001.

|  |  | ARR (n=36) | |  | RHY (n=44) | |  | BS (n=12) | |
| --- | --- | --- | --- | --- | --- | --- | --- | --- | --- |
|  |  | Offset | Post-ictal |  | Offset | Post-ictal |  | Offset | Post-ictal |
| **HPC** | [1–7Hz] | 0.067±0.004 | 0.047±0.003*** |  | 0.079±0.011 | 0.047±0.006*** |  | 0.056±0.006 | 0.047±0.007 |
|  | [8–12Hz] | 0.021±0.002 | 0.011±0.002*** |  | 0.046±0.011 | 0.025±0.006*** |  | 0.015±0.001 | 0.009±0.002*** |
|  | [13–25] | 0.006±0.002 | 0.003±0.001*** |  | 0.017±0.004 | 0.034±0.009** |  | 0.005±0.001 | 0.002±0.001*** |
|  |  |  |  |  |  |  |  |  |  |
| **SN** | [1–7Hz] | 0.011±0.002 | 0.007±0.001*** |  | 0.014±0.002 | 0.011±0.002* |  | 0.017±0.002 | 0.013±0.001* |
|  | [8–12Hz] | 0.003±0.001 | 0.002±0.001*** |  | 0.004±0.001 | 0.003±0.001*** |  | 0.004±0.001 | 0.003±0.001*** |
|  | [13–25] | 0.0006±0.002 | 0.0003±0.001*** |  | 0.001±0.001 | 0.0006±0.001*** |  | 0.0008±0.001 | 0.0005±0.003*** |
|  |  |  |  |  |  |  |  |  |  |
| **HPC/SN** | [1–7Hz] | 0.53±0.014 | 0.53±0.012 |  | 0.53±0.020 | 0.55±0.019 |  | 0.46±0.011 | 0.56±0.019*** |
|  | [8–12Hz] | 0.47±0.011 | 0.52±0.013** |  | 0.53±0.021 | 0.55±0.024 |  | 0.050±0.02 | 0.048±0.015 |
|  | [13–25] | 0.51±0.012 | 0.53±0.008 |  | 0.54±0.014 | 0.54±0.011 |  | 0.46±0.016 | 0.53±0.008*** |
