# Supplementary material for: Seizure onset and offset pattern determine the entrainment of the cortex and substantia nigra in the nonhuman primate model of focal temporal lobe seizures
Source: PLoS One. 2024 Aug 28;19(8):e0307906. doi: 10.1371/journal.pone.0307906 (PMC11356443; doi:10.1371/journal.pone.0307906)
Supplement: S5 Table — (DOCX) [file pone.0307906.s006.docx]

S5 Table: Mean ± SEM values obtained before, at the beginning and the end of the seizures in the SI for LAF and HAS onset patterns. Statistical comparison performed with a Friedman repeated test and Dunnett’s for post hoc comparison with the values preceding the seizures, *<0.05, **<0.01, ***<0.001. Comparisons between LAF and HAS seizures were performed with a Mann-Whitney Rank Sum test (# <0.05). Statistical values were corrected for multiple comparison using Bonferroni correction.

|  |  | LAF (n=36) | |  |  | HAS (n=15) | |
| --- | --- | --- | --- | --- | --- | --- | --- |
|  |  | Pre-ictal | Onset |  |  | Pre-ictal | Onset |
| SI | [1–7Hz] | 0.024±0.002 | 0.033±0.004 ** |  |  | 0.012±0.002### | 0.016±0.004## |
|  | [8–12Hz] | 0.021±0.003 | 0.026±0.003 |  |  | 0.012±0.002# | 0.012±0.003## |
|  | [13–25] | 0.004±0.001 | 0.005±0.001* |  |  | 0.002±0.001## | 0.003±0.001## |
|  |  |  |  |  |  |  |  |
| HPC/SI | [1–7Hz] | 0.46±0.01 | 0.53±0.01*** |  |  | 0.46±0.02 | 0.52±0.02* |
|  | [8–12Hz] | 0.44±0.01 | 0.43±0.01 |  |  | 0.43±0.02 | 0.41±0.01 |
|  | [13–25Hz] | 0.44±0.01 | 0.43±0.01 |  |  | 0.43±0.02 | 0.42±0.01 |
