# Supplementary material for: Seizure onset and offset pattern determine the entrainment of the cortex and substantia nigra in the nonhuman primate model of focal temporal lobe seizures
Source: PLoS One. 2024 Aug 28;19(8):e0307906. doi: 10.1371/journal.pone.0307906 (PMC11356443; doi:10.1371/journal.pone.0307906)
Supplement: S6 Table — (DOCX) [file pone.0307906.s007.docx]

S6 Table: Mean ± SEM values obtained before, at the beginning and the end of the seizures in the SI for LAF and HAS onset patterns. Statistical comparison performed with a Friedman repeated test and Dunnett’s for post hoc comparison with the values preceding the seizures, *<0.05, **<0.01, ***<0.001. Comparisons between LAF and HAS seizures were performed with a Mann-Whitney Rank Sum test (# <0.05). Statistical values were corrected for multiple comparison using Bonferroni correction.

|  |  | ARR (n=35) | |  | RHY (n=9) | |  | BS (n=12) | | |
| --- | --- | --- | --- | --- | --- | --- | --- | --- | --- | --- |
|  |  | Offset | Post-ictal |  | Offset | Post-ictal |  | Offset | Post-ictal | |
| SI | [1–7Hz] | 0.012±0.002 | 0.011±0.002 |  | 0.033±0.007 | 0.028±0.005 |  | 0.022±0.004 | | 0.021±0.003 |
|  | [8–12Hz] | 0.013±0.002 | 0.010±0.001 |  | 0.026±0.004 | 0.020±0.002 |  | 0.024±0.003 | | 0.018±0.003 |
|  | [13–25] | 0.002±0.001 | 0.002±0.001 |  | 0.005±0.001 | 0.004±0.001 |  | 0.004±0.001 | | 0.004±0.001 |
|  |  |  |  |  |  |  |  |  | |  |
| HPC/SI | [1–7Hz] | 0.44±0.01 | 0.46±0.01 |  | 0.56±0.03 | 0.53±0.03 |  | 0.48±0.02 | | 0.47±0.02 |
|  | [8–12Hz] | 0.41±0.01 | 0.43±0.01 |  | 0.44±0.01 | 0.47±0.01 |  | 0.43±0.01 | | 0.45±0.01 |
|  | [13–25Hz] | 0.42±0.01 | 0.43±0.01 |  | 0.44±0.01 | 0.47±0.01 |  | 0.42±0.01 | | 0.44±0.01 |
